# Phenotypic Antimicrobial Susceptibility and Genotypic Characterization of Clinical *Ureaplasma* Isolates Circulating in Shanghai, China

**DOI:** 10.3389/fmicb.2021.724935

**Published:** 2021-10-08

**Authors:** Hongxia Ma, Xuemei Zhang, Xiaoxing Shi, Jun Zhang, Yunheng Zhou

**Affiliations:** ^1^Department of Health Management Medicine, Shanghai East Hospital, Tongji University School of Medicine, Shanghai, China; ^2^Department of Oncology, Shanghai East Hospital, Tongji University School of Medicine, Shanghai, China; ^3^Department of Clinical Laboratory, Shanghai Provincial Crops Hospital of Chinese People’s Armed Police Forces, Shanghai, China; ^4^Department of Clinical Laboratory, Sir Run Run Shaw Hospital Zhejiang University School of Medicine, Hangzhou, China; ^5^Department of Clinical Laboratory, Zhabei Central Hospital of Jing’an District, Shanghai, China

**Keywords:** *Ureaplasma* spp., drug resistance, urogenital tract samples, multilocus sequence typing, resistance mechanism, multidrug resistance

## Abstract

There is a growing global concern regarding the rise of antimicrobial resistance among *Ureaplasma* spp. isolates. However, studies on the antimicrobial susceptibility profiles, resistance mechanisms, and clonality of *Ureaplasma* spp. clinical isolates are still limited and cover only some geographic regions. Firstly, *Ureaplasma* species from the urogenital tracts of patients in Shanghai, China, were isolated by using the culture medium (A8 and 10B broth), and identified the genotype by polymerase chain reaction (PCR). Secondly, the antimicrobial susceptibility tests were determined by using broth microdilution assay. Then, the resistance genetic determinants to fluoroquinolones (FQs), macrolides, and tetracyclines were investigated through PCR/DNA sequencing. Finally, the molecular epidemiology of *Ureaplasma* species was studied by multilocus sequence typing (MLST). Among 258 isolates, *Ureaplasma parvum* (UPA) and *Ureaplasma urealyticum* (UUR) were found in 226 (87.60%) and 32 (12.40%) isolates, respectively. The minimum inhibitory concentrations (MICs) of 258 *Ureaplasma* spp. strains ranged from 0.015 to 64μg/ml for all 11 kinds of antimicrobials. Regardless of species, the isolates were most sensitive to AZI (1.94%), JOS (3.49%), and CLA (4.23%). Among them, there were 39 (15.12%) multidrug-resistant (MDR) strains, including 32 UPA isolates. The resistance rates of UPA to CIP (91.59%), and ROX (36.28%) were significantly higher than those of UUR. Twenty six FQ-resistant isolates had amino acid substitutions in *gyrA* and in *parC* (Ser83Leu). Mutations were detected in genes encoding ribosomal proteins L4 (Thr84Ile) and L22 (Ser81Pro) in macrolide-resistant isolates. *Tet*(M) was found in four UPA isolates. These mutations were mainly found in UPA isolates. Sequence type 1 (ST1) was the predominant ST, which contained 18 isolates. In conclusion, this study showed a higher resistance rate (especially to ROX and CIP), higher substitution rate, and higher MDR rate among UPA strains. The most active antimicrobial agents were AZI, JOS, and CLA. Identifying UPA or UUR in clinical isolates could help clinicians to choose appropriate drugs for treatment. The main resistance mechanisms may involve gene substitution of Ser83Leu in *parC* and Ser81Pro in L22. ST1 was the predominant ST of *Ureaplasma* isolates with MDR to FQs and macrolides in Shanghai, China.

## Introduction

*Ureaplasma* spp. are among the best characterized mycoplasmal bacteria because they are tightly associated with the urogenital tract pathology in humans (Beeton and Spiller, 2017). Owing to their small genome and limited metabolic and biosynthetic capacities, *Ureaplasma* spp. are dependent on an exogenous supply of amino acids and require a close association with host cells for survival. As a result, *Ureaplasma* spp. infections have emerged as a clinical issue in a large group of patients with various diseases, such as nonchlamydial and nongonococcal urethritis (Beeton et al., 2019), prostatitis, cervicitis, as well as pelvic inflammatory disease (Hartmann, 2009). To date, reports have indicated that *Ureaplasma* spp. could be related to adverse neonatal and pregnancy outcomes, including miscarriage and meningitis in newborns (Waites, et al., 2005; Capoccia et al., 2013; Sprong et al., 2020), fatal hyperammonemia in adults (Bharat et al., 2015).

*Ureaplasma* spp. have no cell wall and belong to the class *Mollicutes*; they are characterized by small cell size and genome (Hartmann, 2009). Based on molecular analyses, human *Ureaplasma* spp. are now divided into *Ureaplasma parvum* (UPA) and *Ureaplasma urealyticum* (UUR), which contain at least 14 serovars: UPA (serovars 1, 3, 6, and 14) and UUR (serovars 2, 4, 5, and 7–13; Robertson et al., 2002).

Because *Ureaplasma* spp. lack cell wall and folic acid synthesis capacity, they are naturally insusceptible to β-lactams, sulfonamides, and trimethoprim (Beeton and Spiller, 2017). However, they are generally considered susceptible to tetracyclines, fluoroquinolones (FQs), and macrolides, which interfere with bacterial protein synthesis or DNA replication (Sweeney et al., 2016). In 2011, the Clinical and Laboratory Standards Institute (CLSI) issued the document M43-A: a standard method for performing and validating *in vitro* susceptibility tests for clinical *Ureaplasma* spp. (Clinical and Laboratory Standards Institute, 2011). CLSI M43-A highlights the requirement for standardized media (10B broth or A8 agar). The document lists only five kinds of selected antimicrobials with breakpoints. However, most laboratories lack the capability to cultivate, isolate, and purify fastidious bacteria such as *Ureaplasma* spp. Only a few studies have characterized antimicrobial resistance levels of *Ureaplasma* spp. in China and other countries (Zhang et al., 2014a; Fernández et al., 2016).

We have recently reported the relationship between *Ureaplasma* spp. infection, male infertility, and semen quality, and determined the minimum inhibitory concentrations (MICs) of some antimicrobials for *Ureaplasma* spp. (Zhou et al., 2018b; Wang et al., 2019). Although some studies have evaluated acquired resistance against several of the above-mentioned agents, the detailed mechanisms underlying the resistance and clonality of *Ureaplasma* spp. are still scarce and are limited to several geographic regions (Pereyre et al., 2007; Beeton et al., 2009; Leli et al., 2012; De Francesco et al., 2013; Schneider et al., 2015; Fernández et al., 2016). Therefore, the aim of this study was to study the genotype, the antimicrobial susceptibility; corresponding molecular resistance mechanisms; multidrug resistance (MDR) and sequence type (ST) distribution and clonality of large-scale clinical *Ureaplasma* spp. isolated from the urogenital tracts of patients in Shanghai, China.

## Materials and Methods

### Clinical Specimens and Species Identification

The study was approved by the Ethics Committee of Shanghai Crops Hospital of Chinese People’s Armed Police (No. 20121206). Clinical specimens were collected from the urogenital tracts of patients (33 men and 225 women; medium age, 28.40years; age range, 19–54years) between 2013 and 2014 in three departments (i.e., departments of dermatology, gynecology and obstetrics, and infertility) of the Shanghai Crops Hospital of Chinese People’s Armed Police. The specimens were collected by picking up endocervical cells or vaginal secretion using sterile swabs in women and from urethral secretions or semen in men.

The secretions were inoculated directly on the selective solid culture medium (A8) and the liquid culture medium at 37°C in an atmosphere of 5% CO_2_; the results were observed after 48 and 72h. Liquefied semen was inoculated in the broth medium (100μl) and on the selective agar medium in parallel (20μl). Due to an increase in pH caused by *Ureaplasma* spp. growth, a phenol red indicator was added to media to display a color change. A Leitz Orthoplan microscope was used for direct examination of colonies at 100× magnification. Colonies of *Ureaplasma* spp. were identified presumptively by their characteristic echinus appearance (brown and tiny) on A8 agar in the presence of the Mn indicator (Viscardi, 2010).

### Isolation and Purification

A single *Ureaplasma* spp. colony was placed into 10B broth media under microscope for purification at 37°C. After the color of the liquid medium had changed into light pink or peach after approximately 16–18h of incubation, the liquid medium was measured and adjusted to pH 7.0; then, the purified strains were identified by PCR and stored in multiple aliquots at −80°C in fetal bovine serum in strains bank of Shanghai Crops Hospital. The selective medium agar (A8) and the liquid medium (10B broth) kits and reference strains (ATCC 33175 and ATCC 15488) were provided by Zhongaisheng Hebei Bioscience Technology Inc. (Xingtai, China).

For *Ureaplasma* spp. samples, genomic DNA extraction was performed using a Takara DNA Minikit (Shiga, Japan). These samples were reconfirmed by amplification of the *Ureaplasma* spp.-specific urease gene (a 430-bp DNA product) using U4 and U5 primers. Detection of 284 *Ureaplasma* spp. isolates was done using DNA sequences of the UM-1 primers (UMS125 and UMA226; Blanchard et al., 1993; Teng et al., 1994; Cunningham et al., 2013) and/or 16S-rRNA gene primers UMS 57-UMA 222 for the detection of UPA (327bp), as well as UMS 170-UMA 263 for the detection of UUR (476bp; Kong et al., 2000; Xie et al., 2009).

### Antimicrobial Susceptibility Testing

In this study, 11 antimicrobials were used, including macrolides [erythromycin (ERY), azithromycin (AZI), roxithromycin (ROX), clarithromycin (CLA), josamycin (JOS)], FQs [levofloxacin (LEV), moxifloxacin (MXF), ciprofloxacin (CIP)], and tetracyclines [tetracycline (TET), doxycycline (DOX), and minocycline (MIN)]. All agents (Sigma-Aldrich, St. Louis, MO, United States) were obtained in a powdered form of known purity and were diluted in accordance with their respective manufacturer’s instructions. The MICs were determined in duplicate by broth microdilution performed in 96-well microtiter plates in accordance with the Clinical and Laboratory Standards (CLSI) guidelines and previous reports (Kechagia et al., 2008; Clinical and Laboratory Standards Institute, 2011) using a range of antimicrobial concentrations from 0.0625 to 128μg/ml in 10B broth (Zhong ai sheng). American Type Culture Collection (ATCC) 33,175 (*Ureaplasma urealyticum*) was used as a quality control strain in each broth microdilution assay.

Briefly, we first quantified these organisms for use in MIC determination; then, the frozen culture aliquots were thawed and enriched, where one part was used for colony counting and the rest was stored at 4°C for 48h for MIC. Next, all the antimicrobials were added to 96-well plates in duplicate. The 96-well plates were placed in a biosafety cabinet, dried at room temperature overnight, and then stored at 4°C for use. Then, the microdilution plates were incubated in ambient air at 37°C until the color change of the positive growth control due to the phenol red pH indicator after approximately 16–24h of incubation. The MIC was then determined as the lowest concentration of an antimicrobial agent that did not show any color change at the time of the growth control’s color change. The MIC_50_ and MIC_90_ values were recorded.

### Analysis of Macrolide, FQ and Tetracycline Resistance Mechanisms

Among FQ-resistant isolates (*n*=31; MIC≥32μg/ml), the quinolone resistance-determining regions (QRDRs) of the *gyrA*, *gyrB*, *parC*, and *parE* genes were studied by PCR/DNA sequencing. Primers were designed for amplification, and PCR-amplified DNA was sequenced in both directions to verify the existence by previously described methods (Bébéar et al., 1999, 2000). The *tet*(M) gene was assessed by PCR using Blanchard et al. (1992) primers and cycling conditions described previously in a total of 20 tetracycline-resistant isolates (15 UPA and five UUR isolates) and 59 tetracycline-susceptible isolates (Blanchard et al., 1992). For all macrolide-resistant isolates (*n*=59, 51 UPA and eight UUR isolates), PCR amplification and sequencing were performed for both 23S rRNA alleles and the genes encoding ribosomal proteins L4 and L22 (Pereyre et al., 2007; Beeton et al., 2009; Fernández et al., 2016).

The acquired sequences were analyzed using DNAStar Lasergene 7.1 and were translated into protein sequences by blastx in NCBI. Nucleotide mutations in the 23S rRNA alleles as well as amino acid substitutions in L4 and L22, *gyrA*, *gyrB*, *parC*, and *parE* were identified by comparison with wild-type sequences, i.e., *Ureaplasma parvum* serovar 3 str. ATCC 700970 (GenBank accession numbers AF222894), UPA ATCC 700970, *Ureaplasma urealyticum* serovar 10 str. ATCC 33699 (GenBank accession numbers CP001184; Pereyre et al., 2007; Schneider et al., 2015; Fernández et al., 2016), and UPA serovar 3 str. SV3F4 (GenBank accession numbers AP 014584; Wu et al., 2014).

### Multilocus Sequence Typing

Multilocus sequence typing (MLST; involving the *ftsH*, *rpl22*, *valS*, and *thrS* genes) was performed on a selection of 59 isolates (51 UPA and eight UUR isolates) that were resistant to FQs and macrolides as well as partially susceptible to tetracyclines (Fernández et al., 2016). Population structures of 59 *Ureaplasma* spp. isolates were predicted by eBURST. All the STs were analyzed.

### Statistical Analysis

Data analysis was performed using SPSS, version 21. The Chi-square test was used for comparison of qualitative variables. *p*<0.05 was considered statistically significant.

## Results

### Species Identification and Prevalence of Resistance in *Ureaplasma* spp.

Of the 258 tested specimens, 226 (87.60%) were only positive for UPA and 32 (12.40%) were only positive for UUR (Table 1). There was no significant difference in the distribution of *Ureaplasma* spp. between the two genders (*p*>0.05). More than 40% of all the *Ureaplasma* spp. were isolated from patients aged 19–25years. There was no significant difference in the distribution of UPA and UUR isolates among the age groups (*p*>0.05; Table 2).

**Table 1:** Distribution of *Ureaplasma* spp. species isolated from 258 cases between female and male.

	UPA (N %)	UUR (N %)	Total (N %)
Female	196 (87.11%)	29 (12.89%)	225 (87.21%)
male	30 (90.91%)	3 (9.09%)	33 (12.79%)
Total	226 (87.60%)	32 (12.40%)	258 (100%)

UPA, *Ureaplasma parvum*; UUR, *Ureaplasma urealyticum*.

**Table 2:** The distribution of *Ureaplasma* spp. of 258 cases from the urogenital tracts of patients among different age groups.

Age group (range, years)	UPA (N %)	UUR (N %)	Total (N %)
19–25	98 (88.29%)	13 (11.71%)	111 (43.02%)
26–30	63 (86.30%)	10 (13.70%)	73 (28.29%)
31–35	31 (93.94%)	2 (6.06%)	33 (14.44%)
>35	34 (82.93%)	7 (17.07%)	41 (16.55%)
Total	226 (87.60%)	32 (12.40%)	258 (100%)

UPA, *Ureaplasma parvum*; UUR, *Ureaplasma urealyticum*.

The detailed results for the MIC range, MIC_50_ and MIC_90_ values, and the antimicrobial resistance of *Ureaplasma* spp. (UPA and UUR) are shown in Table 3. The MICs of 258 *Ureaplasma* spp. strains ranged from 0.015 to 64μg/ml for all 11 kinds of antimicrobials *in vitro*. Among them, ciprofloxacin had the least effective activity (MIC_50_ of 16μg/ml and MIC_90_ of 64μg/ml) against *Ureaplasma* spp. CLA was the most active antimicrobial with an MIC_50_ of 0.125μg/ml and MIC_90_ of 0.5μg/ml. ERY was the least effective among macrolides (MIC_50_ of 2μg/ml and MIC_90_ of 16μg/ml). DOX was the most active tetracycline, with MIC_50_ of 0.25μg/ml and MIC_90_ of 2μg/ml.

**Table 3:** Antimicrobial *in vitro* resistance profiles of 258 clinical *Ureaplasma* spp. isolates.

	ERY N (%)	AZI N (%)	ROX N (%)	CLA N (%)	JOS N (%)	LEV N (%)	MXF N (%)	CIP N (%)	TET N (%)	DOX N (%)	MIN N (%)
UPA	39 (17.26)	4 (1.77)	82 (36.28)	9 (3.98)	6 (2.65)	167 (73.89)	61 (27.00)	207 (91.59)	39 (17.2)	22 (9.73)	18 (7.96)
UUR	6 (18.75)	1 (3.2)	3 (9.37)	3 (9.37)	3 (9.37)	21 (65.63)	11 (34.37)	25 (78.12)	6 (18.75)	7 (21.87)	6 (18.75)
*p* value	0.806	-	0.002	-	-	0.395	0.403	0.027	0.806	0.042	-
Total	45 (17.44)	5 (1.94)	85 (32.95)	12 (4.23)	9 (3.49)	188 (72.87)	72 (27.91)	232 (89.92)	45 (17.44)	29 (11.24)	24 (9.30)
MIC range	0.03–64	0.015–16	0.03–64	0.015–16	0.015–16	0.03–64	0.03–64	0.03–64	0.015–16	0.015–16	0.015–16
MIC_50_	2	1	1	0.125	0.125	8	1	16	0.5	0.25	0.5
MIC_90_	16	4	8	0.5	1	64	16	64	8	2	4

According to the CLSI, the top three drug resistance rates of *Ureaplasma* spp. were those to CIP (89.92%), LEV (72.87%), and ROX (32.95%). Among them, the drug resistance rates of UPA were as follows: CIP (91.59%), and ROX (36.28%); they were significantly higher than those of UUR. The most active antimicrobial agents were AZI (1.94%), JOS (3.49%), CLA (4.23%), and MIN (9.30%). Of the 258 *Ureaplasma* spp. strains, there were 77(29.84%) isolates uniformly resistant to ROX and CIP, and 39(15.12%) MDR isolates; both of them were dominated by UPA strains (Table 4).

**Table 4:** Occurrence of ROX-CIP and MDR among *Ureaplasma* spp.

*Ureaplasma* spp. (N %)	ROX-CIP	MDR	Total
UPA	67 (29.65%)	32 (14.16%)	226 (87.60%)
UUR	10 (31.25%)	7 (21.87%)	32 (12.40%)
Total	77 (29.84%)	39 (15.12%)	258 (100%)

ROX-CIP: both resistant to roxithromycin (ROX) and ciprofloxacin (CIP). MDR, multi-drug resistance: resistant to at least one agent in≥3 antimicrobial categories: macrolides, tetracyclines, and fluoroquinolones. UPA, *Ureaplasma parvum*; UUR, *Ureaplasma urealyticum*.

### Molecular Characterization of Antimicrobial Resistance

In the comparison with the relative DNA and protein sequences of reference strains, none of the isolates possessed mutations in 23S rRNA. A Thr84Ile mutation was detected in the ribosomal protein L4 of one roxithromycin-resistant UPA isolate. A Ser81Pro mutation was detected in the ribosomal protein L22 of seven *Ureaplasma* spp. isolates (five roxithromycin-resistant UPA isolates, one erythromycin-resistant UPA isolate, and an UPA isolate resistant to roxithromycin and erythromycin); the latter also had another mutation, Lys91Asn in the protein L22.

The comparison between the QRDR DNA sequences of reference strains and the QRDRs of FQ-resistant isolates (*n*=31; MIC≥32μg/ml) revealed no mutations in *gyrB* or *parE* in any of the isolates. However, *gyrA* and *parC* quinolone resistance-associated mutations were found. One FQ-resistant UPA isolate showing a levofloxacin MIC of 32μg/ml harbored a Val120Phe mutation in *gyrA*, but no QRDR mutations were found in *gyrA* of FQ-resistant UUR isolates. The most frequent mutation was Ser83Leu in *parC*, which was present in 22 (88%) and three (50%) of the FQ-resistant UPA and UUR isolates, respectively. Among 32 MDR UPA isolates, there were 14 cases of mutation in *parC* S83L (Ser83Leu); 2 isolates with Ser83Leu (Ser81Pro) and 1 isolates with *Tet*(M) Screening of the *tet*(M) gene, which is associated with tetracycline resistance, was performed among 79 isolates. Two tetracycline-resistant UPA isolates were positive for this gene, but two tetracycline-susceptible UPA isolates also harbored this gene.

### Multilocus Sequence Typing

Among the 59 *Ureaplasma* spp. isolates resistant to FQs and macrolides, the *ftsH* gene and *thrS* gene, with the highest discriminatory power, had 10 alleles and 8 alleles, respectively, while the *rpL22* gene and *valS* gene had four and three alleles, respectively.

**Figure 1:**
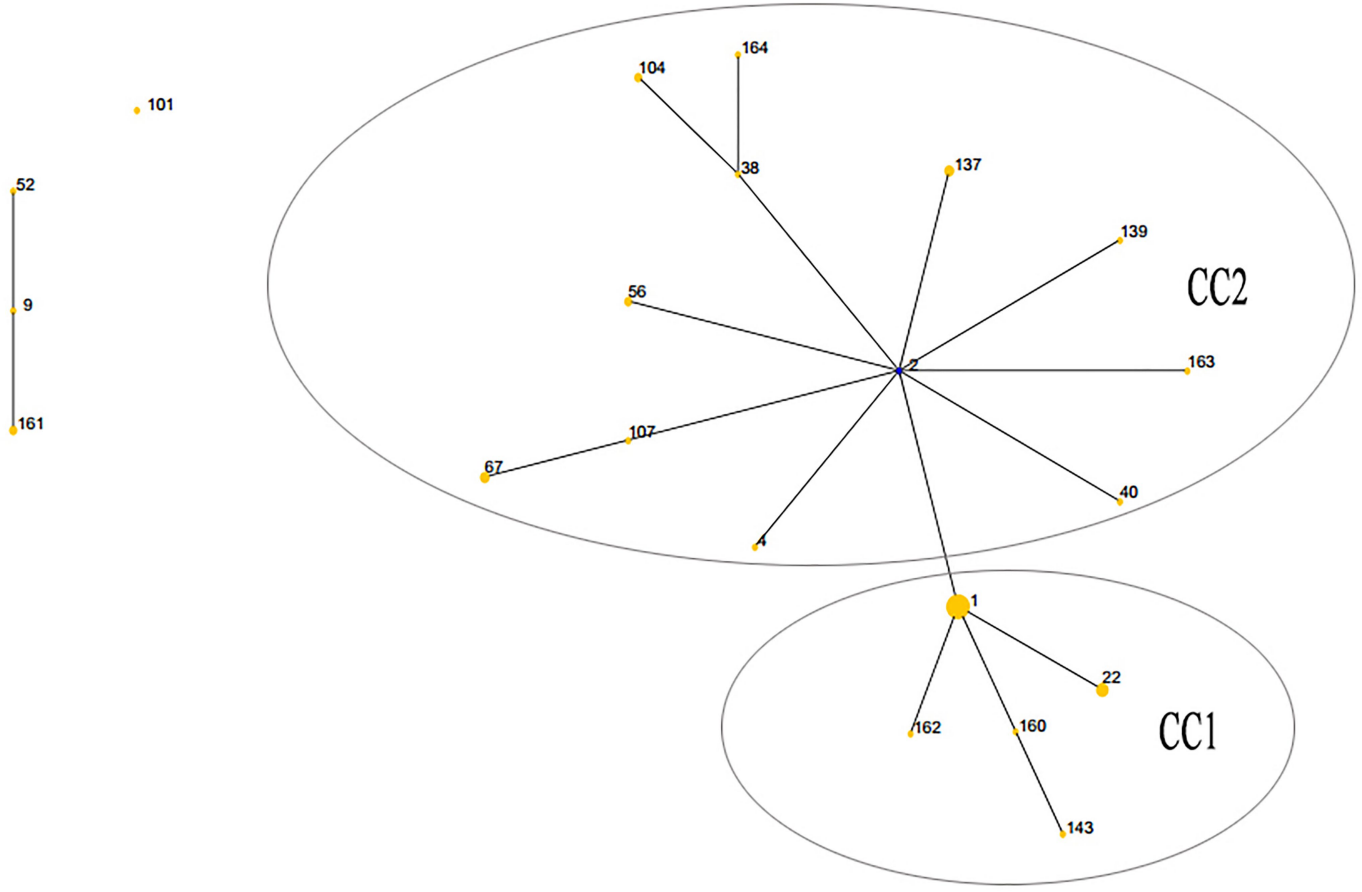
Population structures of 59 *Ureaplasma* spp. isolates resistant to quinolones and macrolides predicted by eBURST. CC, clonal complex.

The lineage of the 21 STs in 59 strains was analyzed, and two major clonal complexes (CC1 and CC2) were revealed by the eBURST package. ST2 was the predicted ancestor, highlighted in the blue circle, while the other STs are displayed in the yellow circle. The size of the circle reflects the number of isolates in the relevant ST, and STs are represented as numbers. Twenty one STs appeared among the 59 clinical isolates. ST1 was the predominant ST, which contained 18 UPA isolates. CC1 contained the vast majority of 17 STs in 44 isolates, while the remaining four STs (ST9, ST52, ST101, and ST161) were singletons (Figure 1).

## Discussion

In recent years, many questions regarding the role of *Ureaplasma* spp. as human pathogens, therapeutic strategies, and the antimicrobial susceptibility of individual species remain open. In this study, we examined the antimicrobial susceptibility, resistance mechanisms, and ST distribution of contemporary 258 clinical *Ureaplasma* spp. isolated from 2013 to 2014 in Shanghai, China. Few previous studies have focused on the individual UPA and UUR distributions in clinical *Ureaplasma* spp. samples (Kawai et al., 2015; Schneider et al., 2015; Yang et al., 2020). In our study, the positive rate of UPA among clinical *Ureaplasma* spp. isolates of the urogenital tract was 87.60%, similar to a recent estimate obtained by Fernández et al. (2016) in the United States. Studies of the frequencies of individual species may be useful for further analyses of their clinical characteristics.

The continuous increase in the prevalence of *Ureaplasma* spp. raised questions regarding their antimicrobial resistance (Zeng et al., 2016; Zhou et al., 2018a). Here, we analyzed the susceptibility of *Ureaplasma* spp. to 11 kinds of antimicrobial drugs. As expected, UPA and UUR had low resistance to most macrolides and tetracyclines, while their resistance to LEV (72.87%) was very high. While the resistance to macrolides is less than 5% in most countries, our study showed a higher rate of ERY (17.44%) and ROX (32.95%) resistance among clinical strains from Shanghai. Additionally, our results significantly differed from those of previous reports. For instance, Fernández et al. have reported a lower resistance rate to LEV (6%) and no macrolide resistance among the 250 clinical isolates in the United States (Fernández et al., 2016). Some domestic studies of *Ureaplasma* spp. strains indicated a 20.09% resistance rate to LEV (Zhu et al., 2012), less than 10% to macrolides (Song et al., 2014), and 76.9% to LEV (Song et al., 2015). In addition, *Ureaplasma* spp. exhibited high resistance to CIP (100%) and to ERY (100% in Tunisia and 80% in South Africa; Redelinghuys et al., 2014; Boujemaa et al., 2020).

Higher resistance rates to ERY, ROX, and LEV may be attributed to the following four factors. First, our study population (87.21% women with border age distribution) may have had higher antimicrobial exposure (especially to macrolides and FQs) and higher resistance levels. Kobayashi et al. found that each age group over 30years had significantly higher odds of receiving FQs for treatment compared with women between 18 and 29years (Kobayashi et al., 2016). However, in Europe and the United States, the macrolide resistance rates of *M. pneumoniae* are substantially lower than those in Asia (Waites et al., 2017). Second, these overall figures indicate that resistance rates vary significantly among countries; in some instances, there are even dramatic differences within the same country, which may be related to the different strategy or preference of antimicrobials usage in different areas (Yang et al., 2020). Third, another explanation for this variation may be related to the shortcomings of different Mycoplasma testing kits and the reference broth microdilution method currently used to estimate the antimicrobial susceptibility of *Ureaplasma* spp. Schneider et al. reported that there was conflicting results from the IST 2 kit and standard broth microdilution was observed for CIP and AZI (Schneider et al., 2015). Namely, the Mycoplasma IST2 assay identifies a number of false positives and does not conform to the approved CLSI guidelines (Beeton et al., 2009, 2015). The accuracy of some reports is still questionable since there are no standardized methods for *in vitro* susceptibility testing and no MIC interpretive breakpoints were designated before 2011. Of note, acidic pH and incubation time affect erythromycin MICs (Kenny and Cartwright, 1993).

Furthermore, mutations, biofilm formation, and the distribution pattern of species or serovars of *Ureaplasma* spp. can also lead to this phenomenon. The top three drug resistance rates of *Ureaplasma* spp. were those to CIP, LEV, and ROX. Among CIP and ROX, the drug resistance rates of UPA were significantly higher than those of UUR. The drug resistance rates to AZI, CLA, JOS, and MIN were very low (less than 10%) regardless of *Ureaplasma* spp.

The relationship and the molecular mechanism between the antimicrobial susceptibility and genotype of *Ureaplasma* spp. have seldom been reported (Song, 2019). Valentine-King and Brown (2017) reported a trend to higher MIC of UUR compared with UPA. The resistance rates were different for different kinds of antimicrobial between UUR and UPA (Fernández et al., 2016). Therefore, analyses of resistance rates of *Ureaplasma* spp. require more data to properly explain and interpret the observed trends.

In this study, we also studied *Ureaplasma* spp. resistance mechanisms to the above-mentioned drugs. As described previously, FQ resistance has been attributed to mutations in *gyrA*, *gyrB*, S83L, S83W, and S84P mutations in the *parC* genes and *parE* genes (Pereyre et al., 2007; Beeton et al., 2015; Kawai et al., 2015; Schneider et al., 2015; Fernández et al., 2016; Valentine-King and Brown, 2017; Meygret et al., 2018). A sequence analysis of the QRDRs revealed that the resistance mechanism of FQ-resistant isolates involves mutations at position 248 (C-T) of *parC*, which leads to a substitution of Ser83 by Leu, followed by a Val120Phe substitution, which was mostly found in UPA isolates. As in previous studies, Beeton et al. (2009) also found that about 80% of isolates were UUR, and the *ParC* Ser83Leu mutation was largely found in all FQ-resistant *Ureaplasma* spp. We found that three FQ-resistant UPA isolates and three FQ-resistant UUR isolates among 31 *Ureaplasma* spp. isolates with high MICs (≥32μg/ml) for LEV, MXF and CIP did not harbor QRDR mutations, suggesting the existence of other undescribed resistance mechanisms in these isolates.

Macrolide resistance has been related to mutations in domain V of 23S rRNA, ribosomal proteins L4 and L22, similar to the mechanism underlying *Mycoplasma pneumoniae* resistance (Schneider et al., 2015; Song et al., 2015, 2019). The macrolide resistance of *Ureaplasma* spp. is still a controversial issue. The A2066G (A to G) mutation and C2243N (T or C) mutation in the 23S rRNA may be associated with *Ureaplasma* spp. macrolide resistance in the Chinese population (Meng et al., 2008; Song et al., 2019). We analyzed the aforementioned resistance traits for 59 *Ureaplasma* spp. isolates showing drug resistance to macrolides; while none of the isolates possessed mutations in the 23S rRNA, we found a mutation in the L4 protein gene and two mutations of the L22 protein gene in eight macrolide-resistant UPA isolates. Yang et al. (2020) reported double alterations (A121S and T141I) or a single mutation (D66N) in L22 within five strains, including one sample harboring an S21A substitution in L4 at the same time. In addition, Pereyre et al. detected two mutations (Lys91Asn and Ala94Asp) in the ribosomal protein L22 and three mutations (Thr70Lys, Gly71Val, and Trp65Arg) in the ribosomal protein L4 (Pereyre et al., 2007); the Lys91Asn mutation in L22 of an erythromycin-resistant UPA isolate was also found in one UPA isolate resistant to erythromycin and roxithromycin and harboring another mutation, Ser81Pro, in our study. The most resistance mechanism to macrolides included the gene mutations in L4 and L22 ribosomal proteins.

The presence of *tet*(M) has been reported in both tetracycline-susceptible and -resistant *Ureaplasma* spp. isolates. In this study, the prevalence of *tet*(M) gene (5.1%, 4/79) was lower than in other reports (Kenny and Cartwright, 1993; Teng et al., 1994; Pereyre et al., 2007; Kotrotsiou et al., 2015). We also found that the *tet*(M) gene may not be a specific indicator of tetracycline-resistant *Ureaplasma* spp. isolates.

In this study, uniform resistance to ROX and CIP was the most frequently observed pattern (29.84%) among *Ureaplasma* spp. isolates; an elevated MDR rate among *Ureaplasma* spp. (15.12%) was detected, particularly among UPA isolates. Zhu et al. (2016) reported only 1.08% MDR of *Ureaplasma* spp. in Nanjing, China, while Boujemaa et al. found an elevated MDR rate (37.62%) with 60.71% of UUR strains and 28.76% of UPA being MDR (Boujemaa et al., 2020). To the best of our knowledge, this is the first report of a higher MDR rate of *Ureaplasma* spp. isolates in Shanghai, China.

To study the molecular epidemiology and population structure of *Ureaplasma* spp., an MLST approach based on four housekeeping genes (*ftsH*, *rpL22*, *valS*, and *thrS*) was first developed by Zhang et al. (2014b) who used 14 serovar reference strains and 269 clinical strains. ST1 and ST22 were the predominant STs and contained 68 and 70 isolates, respectively. Zhang et al. (2014a) also found that isolates of CC1 were UPA and those of CC2 were UUR. CC2 was found more often in symptomatic patients with vaginitis, tubal obstruction, and cervicitis. In addition, two recent studies evaluated the molecular epidemiology of clinical *Ureaplasma* spp. isolates in the United States and Switzerland, making reference to the research of Zhang et al.; these studies suggested the association of molecular epidemiology of *Ureaplasma* spp. isolates with pathogenicity or spread of antimicrobial resistance (Schneider et al., 2015; Fernández et al., 2016). We found that ST1 (18 UPA isolates) was the predominant ST among 59 MDR isolates, followed by ST22 (5 UPA isolates). Among 18 ST1 UPA isolates, there was higher MDR rate (8 cases, 44.44%) and the mutations found as the following: *parC* gene (4 cases), GyrA/*parC* (one case), and L22: S81P (one case). ST1 was the main clone both in Hangzhou (Zhang et al., 2014a) and Shanghai, which may hypothesize that ST1 can be associated with spread of the urogenital tracts of different populations or the antimicrobial resistance. MLST is a well-accepted way for illustrating the diversity and population structure of different bacterial species compared to any other molecular subtyping method. As the strain numbers of STs were very limited, it was difficult to draw any decisive conclusions on any possible correlations between STs and pathologies or molecular resistance mechanisms of *Ureaplasma* spp.

In conclusion, using a large number of clinical isolates, which were mainly obtained from women, this study addressed the lack of data in China related to the antimicrobial susceptibility of *Ureaplasma* spp. The prevalence of antimicrobial resistance in *Ureaplasma* spp., especially that to ERY, ROX, and LEV, is markedly higher in China than in European countries. The most active antimicrobial agents were AZI, JOS, and CLA. Identifying UPA or UUR in clinical isolates could help clinicians to choose appropriate drugs for treatment. For UPA in particular, ERY, ROX, and FQs should be used with caution due to the relatively high rate of MDR. ST1 was the predominant ST of *Ureaplasma* isolates with MDR to FQs and macrolides in Shanghai, China. The main resistance mechanisms may involve gene substitution of Ser83Leu in *parC* and Ser81Pro in L22, in addition to other, undescribed mechanisms. One of the limitations of this study is that it did not provide sufficient data to better understand the epidemiology of *Ureaplasma* spp. in China. Future work should extend our investigations to address the resistance mechanisms related to the spread of antimicrobial resistance, particularly MDR strains, and the difference in drug resistance in different *Ureaplasma* spp.

## Data Availability Statement

The datasets presented in this study can be found in online repositories. The names of the repository/repositories and accession number(s) can be found at: https://www.ncbi.nlm.nih.gov/genbank/MZ700119-MZ700176 for ftsH, MZ710758 – MZ710815 for valS, MZ710816 – MZ710874 for rpL22, MZ710875 – MZ710931 for thrS.

## Ethics Statement

The studies involving human participants were reviewed and approved by the Ethics Committee of Shanghai Crops Hospital of Chinese People’s Armed Police. Written informed consent for participation was not required for this study in accordance with the national legislation and the institutional requirements.

## Author Contributions

HM, JZ, and YZ contributed to conception and design of the study. HM, XZ, XS, and YZ performed the experimental studies and analysis. HM, XZ, and YZ wrote the section of the manuscript. All authors contributed to the article and approved the submitted version.

## Funding

This work was financially supported by the Key Programs of Science and Technology Commission Foundation of Changning District, Shanghai (CNKW2016Z05), the Jiangxi Provincial Health Technology Project (20202114), the National Natural Science Foundation of China (NSFC81370047), and Natural Science Foundation of Shanghai (13ZR1449700).

## Conflict of Interest

The authors declare that the research was conducted in the absence of any commercial or financial relationships that could be construed as a potential conflict of interest.

## Publisher’s Note

All claims expressed in this article are solely those of the authors and do not necessarily represent those of their affiliated organizations, or those of the publisher, the editors and the reviewers. Any product that may be evaluated in this article, or claim that may be made by its manufacturer, is not guaranteed or endorsed by the publisher.
